# Controlling the disease in MYH-associated polyposis

**DOI:** 10.1186/1897-4287-9-S1-P19

**Published:** 2011-03-10

**Authors:** Lisa LaGuardia, Margaret O’Malley, Carol Burke, Matthew Kalady, James Church

**Affiliations:** 1 The Sanford R. Weiss, M.D. Center for Hereditary Colorectal Neoplasia, Department of Colorectal Surgery, Digestive Disease Institute, 9500 Euclid Ave., Cleveland Clinic, Cleveland, Ohio, 44195, USA

## Background

MYH-Associated Polyposis (MAP) is an autosomal recessive condition caused by bi-allelic mutations in *MYH*. Individuals with MAP tend to develop numerous polyps in their colon and rectum and have an increased risk of developing colorectal cancer. Recommendations for MAP treatment vary in the literature ranging from frequent surveillance colonoscopy to prophylactic surgery depending on polyp burden. The aim of this study was to report the management and outcome of a single institution series of patients with MAP.

## Methods

Patients with biallelic mutations in *MYH* were accrued over 23 years from a query of a comprehensive polyposis database using Cologene© software. Demographics, family history, upper and lower endoscopy frequency, polyp burden, and cancer data, and treatment were recorded.

## Results

Thirty-four patients from 26 families with MAP were included. There were 24 cancers in 14 patients. Four of the patients had multiple cancers (14 total), each with a rectal cancer plus a more proximal cancer. 3 patients had more than 2 colorectal cancers. Of the 24 colorectal cancers, 10 (42%) were right sided and 14 (58%) were left sided. Most cancers (90%) were stage I or II and 10% were stage III as shown in Table 1.

**Table 1:** Demographic Details

	Synchronous (N=4)	Solitary (N=10)	No Cancer (N=20)
Gender	M – 50% F - 50%	M – 60% F – 40%	M – 90% F – 10%
Age	Mean 40.8	Mean 51	Mean 54
Family History	3/4 yes	4/10 yes (one unknown)	6/20 yes

All patients also had colorectal adenomas. Median polyp number is 20 (range 1-115) for patients with solitary cancer and for multiple cancers median was 100 (range 50-120) (Table 2).

**Table 2:** Cancer Details

	Synchronous (N=4)	Solitary (N=10)	No Cancer (N=20)
Number of polyps	Median 100 (50-200)	Median 20 (1-115)	Median 50 (15-116) Surgery Median 17 (4 -100) No Surgery

Cancer Stage Stage I Stage II Stage III	1 (25%) 2 (50%) 1 (25%)	5 (50%) 4 (40%) 1 (10%)	

Location	100% Right side 50% Left side 100% Rectum	80% Colon 20% Rectum	

All patients with cancer had a resection. Four patients underwent segmental colectomy, 5 underwent a colectomy with ileorectal anastomosis, and 5 were treated with total proctocolectomy (4 with ileal anal pouch and one with an end ileostomy). There have been no deaths from colon or rectal cancer and no recurrence at a mean follow-up of 96 months.

## Conclusion

MAP is associated with an increased risk of colorectal cancer, but appropriate surveillance and surgical intervention prevents cancer-related deaths.

